# Supplementary material for: A Controlled Clinical Trial on the Effects of Aquatic Exercise on Cognitive Functions in Community-Dwelling Older Adults
Source: Brain Sci. 2024 Jul 13;14(7):703. doi: 10.3390/brainsci14070703 (PMC11275130; doi:10.3390/brainsci14070703)
Supplement: Supplementary file 1 [file brainsci-14-00703-s001.zip › Additional file 3_Fidelity Checklist_Self-Rating.pdf]

Additional Table 4: Clinician self-rating checklist

**Therapeutic attitude**	Fully agree (4)	Partly agree (3)	Partly disagree (2)	Fully disagree (1)	Not applicable (0)	Comments
1. During the session I have taken into account that the participant is the expert of his/her own life.<br><br>**Optional reflection by the therapist:**<br>It is up to the participant to know how they want to organise their life. As a therapist, I adopt a "non-knowing attitude" and listen with curiosity. I avoid diagnosing problems and do not impose solutions (non-directive approach).						
2. I have taken into account during the session that the participant has individual resources, even if they are not yet aware of them.<br><br>**Optional reflection by the therapist:**<br>I listened attentively and sympathetically and paid attention to success stories in the participant's biography. With the help of questions, I supported the participant in identifying unrecognised resources and skills from their biography (does not apply to the main narrative in the narrative interview).						
3. I have taken into account during the session that my central task is to support the communication of the participant so that identity work can take place.<br><br>**Optional reflection by the therapist:**<br>I was primarily a listener and felt particularly responsible for ensuring that the narrative could take place despite aphasia.						
4. I have taken into account during the session that the participant is doing his/her best and cooperating in his/her own way.<br><br>**Optional reflection by the therapist:**<br>I assume that every person is motivated by something, and it is my job as a therapist to find out what that something is. What the participant says cannot be wrong.						
5. I am convinced that the biographic-narrative work itself can contribute to the development of the participant's identity.<br><br>**Optional reflection by the therapist:**<br>I use questions to encourage the participant to reflect on biographical content.						
6. I followed the participant's narrative throughout the session.<br><br>**Optional reflection by the therapist:**<br>I listened to the participant's stories and noted down narrative cones. I paid attention to what was important to the participant.						

Observable behaviours	Fully agree (4)	Partly agree (3)	Partly disagree (2)	Fully disagree (1)	Not applicable (0)	Comments
1. I did my best to facilitate the communication of the person with aphasia (e.g. by paraphrasing / using sounds / using pictures, pictograms or written language). During the main narrative, however, I held back more so as not to interrupt the narrative flow.						
2. At the end of each session I asked the participant to give feedback and reflect on their own mood.						
3. I have focussed on non-judgemental storytelling.						
4. I only interrupted the participant if it was unavoidable (e.g. because I did not understand something).						
5. I did not correct the participant linguistically.						
6. I could stand breaks.						
7. I put my own opinion aside and did not provide any solutions.						
8. I picked up on disturbances (noise etc.) and only then continued the conversation.						
9. I have taken up statements made by the participant (e.g. by repeating / paraphrasing) and during the main narrative and in the case of immanent questions I have only dealt with those contents that were mentioned by the participant.						
10. The content of the session was customised to the individual participant (e.g. by selecting questions from the guide).						
11. The participant was encouraged by my questions to report biographical content (specific content such as hobbies).						
12. I asked exmanent questions in a way that emphasised the participant's resources.<br><br>**! This item is only applicable from the point at which exmanent questions are used. Before that, it should be disregarded in the assessment!**						
13. I have addressed all three levels of time (past, present and future) today.<br><br>**! This item is only applicable when new topics have been introduced with the help of external questions. Before that, it should be disregarded in the assessment!**						
14. I introduced new topics (e.g. health / illness) in a non-judgemental way so that they could be evaluated individually by the participant.<br><br>**! This item is only applicable once the terms health / illness have been introduced. Before that, it should be neglected in the assessment!**						
15. The expectations and wishes of the participant were taken up by the entire group with a basic attitude of acceptance and without judgement.						

**! This item is only applicable for the group sessions and can be neglected in the evaluation of the individual sessions!**						

**Assessment of basic therapeutic attitude and observed behaviour (max. 68 to 84 points):**<br>If items 12-15 are applicable, the total score should be divided by 84.<br>If three of the items 12-15 are applicable, the total score should be divided by 80.<br>If two of the items 12-15 are applicable, divide the total score by 76.<br>If only one of the items 12-15 is applicable, divide the total score by 72.<br>If none of the items 12-15 are applicable, divide the total score by 68.<br><br>The proportional total value (< 1) can be multiplied by 100.<br>The result describes the percentage of adherence to the *narraktiv* guideline.<br><br>An example of the evaluation of a group session after the introduction of the terms health / illness and new topics:<br>(65 / 84) x 100 = 77,4 %
**Assessment of observable behaviour (max. 44 to 60 points)**<br>If items 12-15 are applicable, the total score should be divided by 60.<br>If three of the items 12-15 are applicable, the total score should be divided by 56.<br>If two of the items 12-15 are applicable, divide the total score by 52.<br>If only one of the items 12-15 is applicable, divide the total score by 48.<br>If none of the items 12-15 are applicable, divide the total score by 44.<br><br>The proportional total value (< 1) can be multiplied by 100.<br>The result describes the percentage of adherence to the *narraktiv* guideline.<br><br>An example of the evaluation of a group session after the introduction of the terms health / illness and new topics:<br>(40 / 60) x 100 = 66,7 %
